# A cost-effectiveness analysis comparing pembrolizumab combined with chemotherapy versus chemotherapy alone for advanced biliary tract cancer: US and China perspectives

**DOI:** 10.1371/journal.pone.0341154

**Published:** 2026-01-22

**Authors:** Chunhua Zhang, Hua Liang, Yanni Qin, Xiaolan Tan, Xiaoqing Su, Xin Tian, Yumei Nong

**Affiliations:** 1 Department of Pharmacy, The Reproductive Hospital of Guangxi Zhuang Autonomous Region, Nanning, Guangxi, People’s Republic of China; 2 Department of Pharmacy, Baise People’s Hospital, Baise, Guangxi, People’s Republic of China; 3 Department of Pharmacy, The People’s Hospital of Chongzuo, Chongzuo, Guangxi, People’s Republic of China; Hokkaido University: Hokkaido Daigaku, JAPAN

## Abstract

**Objective:**

In the KEYNOTE-966 study, the clinical benefits of pembrolizumab plus chemotherapy were demonstrated for patients with advanced biliary tract cancer (BTC). At this point, it is unknown whether this expensive therapy is cost-effective. The purpose of this study was to evaluate the cost-effectiveness of pembrolizumab plus chemotherapy in treating BTC.

**Methods:**

We constructed a partitioned survival model form the perspectives of US and Chinese payers. KEYNOTE-966 was used to obtain the baseline characteristics of the patients as well as their clinical data. Local databases and published literature were used to collect costs and utilities. Costs, life years, quality-adjusted life years (QALYs), incremental cost-effectiveness ratios (ICERs), incremental net health benefits (INHB), and incremental net monetary benefits (INMB) were measured and compared. We conducted sensitivity analyses in order to assess the robustness of the model. Subgroup analyses were also performed.

**Results:**

Pembrolizumab plus chemotherapy is not cost-effective in China at the willingness to pay (WTP) thresholds of $38,258 and $84,866. However, it yielded an additional 0.137 QALYs and an additional $63,864 (ICER $466,340) over chemotherapy alone. In the US, this treatment was not cost-effective, resulting in an improvement in effectiveness of 0.144 QALYs and an increase in overall cost of $141,000 (ICER of $976,925). There were INHBs of −0.616 QALYs and INMBs of -$52,237 for pembrolizumab plus chemotherapy in China if the WTP threshold for QALYs was set at $84,866, and INHBs of −0.796 QALYs and INMBs of -$119,400 when the WTP threshold was set at $150,000 for the US. Through sensitivity analyses, it was demonstrated that the results were stable. The results of the subgroup analysis indicate that better survival properties subgroups were more likely to be cost-effective, although pembrolizumab plus chemotherapy may not be cost-effective for all subgroups.

**Conclusions:**

In the US and China, pembrolizumab plus chemotherapy may not be a cost-effective treatment option. This study provides evidence-based pricing strategies that may benefit decision makers and clinicians as they make clinical decisions. For a better understanding of the impact on budgets and the affordability of care for patients, more evidence is required.

## Introduction

The biliary tract cancer (BTC), consisting of intrahepatic cholangiocarcinoma, extrahepatic cholangiocarcinoma, and gallbladder cancers, is a rare group of heterogeneous and aggressive epithelial cancers [1,2]. Patients with cancers of the intrahepatic or extrahepatic bile ducts and gallbladder generally have poor outcomes [1,2]. As the most common hepatobiliary cancer in the United States (US) after hepatocellular carcinoma, BTC represents approximately 3% of all gastrointestinal malignancies [3]. Most BTC patients are diagnosed at a late stage, resulting in a poor prognosis of only 2% in terms of the 5-year survival rate [4]. Moreover, several inflammatory conditions can lead to biliary tract injuries, including chronic viral hepatitis and cirrhosis [1,5]. There is significant molecular heterogeneity among BTC, which is influenced by the site and cause of the cancer [6,7]. The incidence of BTC appears to be on the rise, despite the fact that it accounts for less than 1% of all new cancer cases worldwide [1,5,8–10]. A rise in intrahepatic cholangiocarcinoma has contributed to an increase in BTC incidence globally [11]. Multiple factors may be responsible for the rise, including misclassification of cholangiocarcinoma, improvements in diagnostic tools, and changes in demographics [12]. There are periodic updates to the International Classification of Disease for Oncology, resulting in changes in the classification of tumors that arise in the liver and those that arise extrahepatically [13].

Advanced BTC have traditionally been treated with systemic chemotherapy. Based on the findings of the randomized phase 3 ABC-02 study, gemcitabine plus cisplatin has been recommended as the standard first-line therapy for advanced BTC [14]. In comparison to gemcitabine monotherapy, mammalian patients treated with gemcitabine plus cisplatin can have improved overall survival (OS) and progression-free survival (PFS). Although intensive study has been conducted, triplet chemotherapy regimens and combination therapies of chemotherapy and targeted therapies have not proven to be more effective than gemcitabine and cisplatin alone [1,15,16]. Combinations based on fluorouracil have modest efficacy, in the course of disease progression [17,18]. It is possible for patients with cancers characterized by specific molecular aberrations, such as FGFR2 fusions, IDH1 mutations, and mismatch repair deficiencies, to benefit from targeted therapies or immune checkpoint inhibitors (ICIs). This is based on results observed in studies conducted in the second-line or later settings [1,19,20]. In addition to regulating the immune system, immune checkpoint proteins are capable of recognizing and destroying cancer cells. Furthermore, ICIs inhibit antitumor immune responses, including programmed cell death protein-1 (PD-1), programmed apoptosis ligand 1 (PD-L1) and cytotoxic T lymphocyte-associated antigen 4 (CTLA-4) [21,22]. Among other causes, BTC are characterized by high levels of heterogeneity caused by mutations in tumor genes, which may be related to neoantigen expression [22]. During immunosuppression, the tumor microenvironment creates a biochemical environment conducive to immunosuppression [23,24]. A recent randomized phase 3 trial, KEYNOTE-966 (NCT04003636), examined the effect of pembrolizumab plus chemotherapy in patients with BTC [25]. The results of KEYNOTE-966 revealed that pembrolizumab plus chemotherapy significantly prolonged OS (hazard ratio [HR] of 0.83 (95% confidence interval [CI]: 0.72–0.95]), with no unexpected immune-related adverse events occurring as a result of the treatment [25].

Despite these encouraging results, cost-effectiveness needs to be considered, as combination therapy including pembrolizumab and chemotherapy is generally more expensive than chemotherapy alone. The purpose of this study was to evaluate the cost-effectiveness of pembrolizumab plus chemotherapy compared with chemotherapy as the first-line therapy in previously untreated BTC patients from the perspectives of US and Chinese payers in order to guide drug pricing. This analysis seeks to provide evidence that can inform reimbursement policies, optimize healthcare resource allocation and support value-based pricing strategies. Given the inherent differences in healthcare systems, and drug pricing between the US and China, our findings will offer critical insights for policymakers striving to balance clinical benefit with economic sustainability.

## Methods

### Study overview

In this study, the Consolidated Health Economic Evaluation Reporting Standards (CHEERS) were followed [26]. Data from a phase 3 global, double-blind, placebo-controlled study were used for this economic evaluation (KEYNOTE-966), in which 1069 target patients were enrolled [25]. KEYNOTE-966, a phase 3 study, was conducted in a randomized, double-blind, and placebo-controlled manner at 175 medical centers located across Asia-Pacific, Europe, North America, and South America. Based on KEYNOTE-966, the participants had to be 18 years or older and were with unresectable, locally advanced or metastatic extrahepatic cholangiocarcinoma, gallbladder cancer, or intrahepatic cholangiocarcinoma; investigator determined Response Evaluation Criteria in Solid Tumors (RECIST) version 1.1, the patient was able to demonstrate disease; the patient had a performance status of 0 or 1 as determined by the investigator; tumor tissue was provided by the patient for biomarker evaluation; they had adequate organ function; and a lifespan exceeding 3 months. Patients who were included received pembrolizumab 200 mg or placebo once every 3 weeks along with gemcitabine 1000 mg/m^2^ and cisplatin 25 mg/m^2^ on days 1 and 8. A constant body surface area (BSA) of 1.86 m² was assumed for dosing calculations, consistent with prior BTC economic evaluations [27]. When the disease progressed or unacceptable toxicity developed, first-line treatments were discontinued; both arms of the study were able to receive subsequent treatment if necessary. The subsequent treatments were derived from KEYNOTE-966 [25]. In response to recommendations from the National Comprehensive Cancer Network (NCCN), we adopted subsequent treatment strategies, owing to the lack of detailed information collected during the preliminary trial [28]. Drug acquisition costs, administration fees, adverse event management costs, follow‑up, and terminal care costs were obtained from published literature, national drug price databases, and official reimbursement lists in China and the United States, with all costs converted to 2023 US dollars using the medical care component of the Consumer Price Index.

### Model construction

In comparing medical costs and clinical outcomes between patients with BTC treated with pembrolizumab plus chemotherapy and those treated with chemotherapy, we used a partitioned survival model [29]. The health states included in this model were PFS, progressed disease (PD), and death. There were deaths in more than 98% of patients in both treatment arms with a 10-year time horizon, which means we considered it a lifetime study [30]. The cycle length was 1-week. Subsequent treatment costs were applied according to the proportion of patients receiving each regimen after progression, and no treatment crossover between arms was assumed unless specified in the trial or guidelines. The model assumed that all patients entered the progression‑free state at baseline, that the proportion of patients in the progression‑free state at time t was taken directly from the PFS curve, the proportion of patients whose disease had progressed at time t was derived by subtracting the proportion in the progression‑free state from the overall survival at time t, and the proportion of patients who had died was calculated as one minus the overall survival at time. This analysis adopted the health care perspectives of third-party payers in China and the US, respectively. We also analyzed the model only PFS state outputs. In China, costs (converted to US dollars) and utilities were discounted at 5%, whereas in the United States, both costs and utilities were discounted at 3% [31,32]. Considering the disparity in socioeconomic development between Chinese regions, ICERs were calculated by using two willingness to pay (WTP) thresholds: gross domestic product (GDP) per capita was three times the value of the per capita GDP of China ($38,258/QALY) for general regions, and three times the GDP of Beijing based on the amount ($84,866/QALY) in 2023 [33]. In the US, the WTP threshold was set at $150,000 [34]. For survival extrapolation, the best‑fitting parametric functions for OS and PFS were applied to project outcomes beyond the observed trial follow‑up to the 10‑year horizon. Extrapolations assumed that hazards followed the chosen parametric form over the remaining time horizon, with no additional treatment‑effect modification beyond the modeled survival curves. Consistency constraints were imposed to ensure that OS was always greater than or equal to PFS at all time points. Alternative parametric forms and time horizons were explored in sensitivity analyses to evaluate the impact of different extrapolation assumptions on model outcomes. In this study, statistical analyses were performed by R (version 4.2.1, 2023, R Foundation for Statistical Computing) with hesim and heemod packages.

### Effectiveness

Through GetData Graph Digitizer (http://getdata-graph-digitizer.com), the time-to-event data of OS and PFS were obtained from the Kaplan-Meier curves of the KEYNOTE-966 study. Moreover, the method of Guyot et al. was used to reconstruct individual patient data (IPD) over the course of the clinical trial [35]. It was found that the number of virtual IPDs was almost and the patients at risk were equal to the initial numbers, resulting in a close representation of digitized KM curves, since they encompassed event and censor times. Parametric function was fitted to these data points based on the following parameters: Weibull, log-normal, log-logistic, exponential, generalized gamma, and Gompertz. Detailed result of parametric function are shown in the S1 Fig and Table 1. We evaluated good-fit using the Akaike Information Criterion (AIC) and the Bayesian Information Criterion (BIC) as well as visual inspection [36]. Combined with appropriate visual effects, a model with a lower AIC and BIC value was likely to provide a better fit [37]. Detailed information regarding the variables of the final survival functions for pembrolizumab plus chemotherapy and chemotherapy can be found in Table 1, and goodness-of-fit is presented in S1 Table. In addition, to ensure the accuracy of the reconstructed IPD using the Guyot method, we performed validation checks against the published Kaplan–Meier curves from KEYNOTE‑966. The reconstructed curves yielded hazard ratios, confidence intervals, and p‑values that were highly consistent with the original trial results (S2 Fig).

**Table 1:** Key model inputs.

Parameter	Value (95% CI)	Distribution	Source
Log-logistic OS survival model of pembrolizumab plus chemotherapy^a^	γ = 1.7679 λ = 0.0185	NA	24
Lognormal PFS survival model of pembrolizumab plus chemotherapy^a^	μ = 3.2319 σ = 1.1380	NA	24
Log-logistic OS survival model of chemotherapy^a^	γ = 1.7565 λ = 0.0216	NA	24
Lognormal PFS survival model of chemotherapy^a^	μ = 3.1082 σ = 1.0149	NA	24
**Costs in the China, $**			
Price of pembrolizumab per 1 mg	26.66 (Range: 21.33 to 30.93)	Gamma	Local database
Price of gemcitabine per 1 mg	0.093 (Range: 0.075 to 0.108)	Gamma	Local database
Price of cisplatin per 1 mg	0.25 (Range: 0.2 to 0.29)	Gamma	Local database
Second-line treatment in pembrolizumab plus chemotherapy arm per cycle	246 (Range: 197–286)	Gamma	24; Local database
Second-line treatment in chemotherapy arm per cycle	319 (Range: 255–370)	Gamma	24; Local database
Cost of best supportive care per cycle	41.82 (Range: 31.37 to 52.28)	Gamma	37
Cost of follow-up and monitoring per cycle	18.39 (Range: 13.79 to 22.99)	Gamma	37
Cost of drug administration per unit	20.3 (Range: 16.24 to 23.55)	Gamma	38
Cost of terminal care per patient^b^	4518 (Range: 3614–5241)	Gamma	39
**Cost of managing AEs (grade ≥ 3)** ^c^			
Pembrolizumab plus chemotherapy	5001 (Range: 4001–5801)	Gamma	38,40,41
Chemotherapy	5116 (Range: 4093–5935)	Gamma	38,40,41
**Costs in the US, $**			
Price of pembrolizumab per 1 mg	55.42 (4 Range: 4.33 to 64.28)	Gamma	42
Price of gemcitabine per 1 mg	0.019 (Range: 0.015 to 0.022)	Gamma	42
Price of cisplatin per 1 mg	0.17 (Range: 0.13 to 0.2)	Gamma	42
Second-line treatment in pembrolizumab plus chemotherapy arm per cycle	1168 (Range: 934–1355)	Gamma	24,42
Second-line treatment in chemotherapy arm per cycle	1562 (Range: 1249–1811)	Gamma	24,42
Cost of best supportive care per cycle	544 (Range: 435–631)	Gamma	43
Cost of follow-up and monitoring per cycle	485 (Range: 388–563)	Gamma	44,45
Cost of drug administration per unit	336 (Range: 269–390)	Gamma	46
Cost of terminal care per patient^b^	17116 (Range: 13693–19855)	Gamma	47,48
**Cost of managing AEs (grade ≥ 3)** ^c^			
Pembrolizumab plus chemotherapy	21316 (Range: 17053–24727)	Gamma	49-51
Chemotherapy	21331 (Range: 17065–24744)	Gamma	49-51
**Health utilities**			
**Disease status utility per year**			
PFS	0.76 (Range: 0.61 to 0.88)	Beta	52
PD	0.68 (Range: 0.54 to 0.79)	Beta	52
Death	0	NA	
**Disutility due to AEs (grade ≥ 3)** ^d^			
Pembrolizumab plus chemotherapy	0.043 (Range: 0.035 to 0.05)	Beta	49,53,54
Chemotherapy	0.039 (Range: 0.031 to 0.045)	Beta	49,53,54
**Other inputs**			
Body surface area, m^2^	1.86 (Range: 1.40 to 2.23)	Normal	26

AE, adverse event; NA, not available; OS, overall survival; PD, progressed disease; PFS, progression-free survival.

^a^Only expected values are presented for these survival model parameters.

^b^Overall total cost per patient regardless of treatment duration.

^c^Calculated as the average cost of toxic effects using weighted frequencies of grade ≥ 3 treatment related adverse events for each treatment arm in the KEYNOTE-966 trial. Costs of individual toxic effects were derived from the literature and include all care required to manage each toxic effect. References for individual toxic effect costs are summarized in S3 Table.

^d^Calculated as the average disutility of toxic effects using weighted frequencies of grade ≥ 3 treatment-related adverse events for each treatment arm in the KEYNOTE-966 trial. Disutilities of individual toxic effects were derived from the literature. References for individual toxic effect disutilities are summarized in S3 Table.

### Cost

Based on health care perspectives and third-party payers in China and the US, respectively, this analysis evaluated only direct medical costs. These included first- and second-line therapeutic strategies, management of adverse events (AEs) related to the drug, routine monitoring and follow-ups, best supportive care and terminal care [38–52]. Our local database or public databases were utilized to obtain drug prices which were inflation adjusted for the year 2023. We chose the most reasonable dosage combination between carboplatin and paclitaxel as there were multiple dosage forms available on the Chinese market. Information on subsequent therapies was derived from KEYNOTE‑966 and NCCN guidelines. The specific agents, their unit costs, and the proportions of patients receiving each treatment in both study arms are summarized in S2 Table. These assumptions were incorporated into the model to capture the impact of post‑progression therapy on overall survival and costs. Grade ≥ 3 AEs were considered only if they occurred with a rate over 3%, and these included neutrophilia, anemia, thrombocytopenia, fatigue and asthenia, and blood count decreases [25]. Based on published articles, the costs and duration of AEs were calculated [39,41,42,50–52]. All the cost variables inputs are shown in Tables 1 and S3.

### Utility

Health technology assessment results indicating a PFS and PD of 0.76 and 0.68 for advanced BTC patients treated with durvalumab and chemotherapy were obtained from the health technology assessment [53]. The disutility value owing to the AEs was extracted from other studies and included in this analysis [50,54,55]. All AEs were assumed to occur during the first cycle [56]. All the utility variable inputs are shown in Tables 1 and S3. These utility estimates were derived from multiple published health technology assessments and cost‑effectiveness studies in advanced BTC and other gastrointestinal malignancies with comparable disease burden. Utility values were assumed to remain constant within each health state and to be independent of treatment arm, except for temporary decrements due to adverse events.

### Base-case analysis

We examined the incremental cost-effectiveness ratios (ICERs) of per additional quality-adjusted life-years (QALYs) gained versus incremental cost. The cost-effectiveness of the treatment was assumed in situations where the ICER was lower than the WTP threshold ($38,258/QALY and $84,866/QALY in China, $150,000/QALY in the US). A calculation was also made for the incremental net monetary benefits (INMB), and the incremental net health benefits (INHB). The following are the formulae used to calculate INHB and INMB: $\text{INHB}(\lambda )=\left(\mu E_{\text{Pembrolizumab}\;\text{plus}\;\text{chemotherapy}}\;-\;\mu E_{Chemotherapy}\right)-\frac{\left(\mu C_{\text{Pembrolizumab}\;\text{plus}\;\text{chemotherapy}}\;-\;\mu C_{Chemotherapy}\right)}{\lambda }=\;\Delta E-\;\frac{\Delta C}{\lambda }$ and $\text{INMB}(\lambda )=(\mu E_{\text{Pembrolizumab}\;\text{plus}\;\text{chemotherapy}}-\mu E_{Chemotherapy}\times \lambda -\left(\mu C_{\text{Pembrolizumab}\;\text{plus}\;\text{chemotherapy}}-\mu C_{Chemotherapy}\right)=\Delta E\;$× λ−ΔC, where μC and μE were the cost and utility of pembrolizumab plus chemotherapy or chemotherapy, respectively, and λ was the WTP threshold [57,58]. In addition, model outputs for only the PFS state were examined.

### Sensitivity analysis

In order to evaluate the robustness of the model, sensitivity analysis was performed. A deterministic sensitivity analysis (DSA) was performed by adjusting each variable within the 95% CIs or assuming reasonable ranges of values for the base-case values (±20%). The probability sensitivity analysis (PSA) was performed with 10,000 Monte Carlo simulations. The cost was distributed using a gamma distribution, probability, proportion, and utility were distributed using beta distributions, and the surface area of the body was distributed using a normal distribution [56]. Various WTP thresholds were selected to analyze the cost-effectiveness of each regimen. Cost-effectiveness acceptability curves were employed to calculate the cost-effectiveness of each treatment.

### Subgroup analysis

ICERs were calculated based on KEYNOTE-966 HRs for OS for each subgroup used in the subgroup analysis. Various subgroups of patients with different ages, sex, geographical regions, Eastern Cooperative Oncology Group (ECOG) performance status scores, smoking status, antibiotic use within 1 month of study start, site of origin, disease status, biliary stent or drain, previous chemotherapy, and PD-L1 combined positive score. As there were insufficient data for the OS and PFS subgroups, we assumed proportional hazards for all the other subgroups.

## Results

### Base‑case analysis results

The results of the base-case analysis are illustrated in Table 2. In China and the US, the cost of pembrolizumab plus chemotherapy was higher than that of chemotherapy alone ($85,480 versus $21,616, $297,282 versus $156,281). China and the US reported 0.211 life years and 0.137 QALYs or 0.144 QALYs improvement with the pembrolizumab plus chemotherapy compared with chemotherapy alone. Based on comparison of the ICER between pembrolizumab plus chemotherapy and chemotherapy alone, the ICER for pembrolizumab plus chemotherapy was $466,340 per QALY (> WTP of $84,866) and $976,925/QALY (> WTP of $150,000) for the China and the US, respectively. As a result, pembrolizumab was not cost-effective in China and the US for the treatment of BTC. In addition, we performed an alternative analysis focusing only on PFS. In China and the US, the ICER for pembrolizumab plus chemotherapy compared with that of chemotherapy alone was $535,405/QALY and $1,118,607/QALY. Moreover, for pembrolizumab plus chemotherapy, the INHB was −0.616 QALYs and the INMB was -$52,237 when the WTP threshold for China was $84,866 per QALY, and for US, the INHB was −0.796 QALYs and the INMB was -$119,400.

**Table 2:** Summary of cost and outcome results in the base-case analysis.

Factor	Pembrolizumab plus chemotherapy	Chemotherapy	Incremental change^a^
**Cost, $ in China**			
Drug	74,592	11,292	63,300
Nondrug^b^	10,888	10,324	564
Overall	85,480	21,616	63,864
**Cost, $ in US**			
Druga	172,816	43,948	128,868
Nondrug^b^	124,466	112,333	12,133
Overall	297,282	156,281	141,000
**Life-years**			
Progression-free survival	0.907	0.726	0.181
Overall	1.647	1.436	0.211
**QALYs, in China**			
Progression-free survival	0.644	0.525	0.119
Overall	1.090	0.953	0.137
**QALYs, in US**			
Progression-free survival	0.660	0.534	0.126
Overall	1.127	0.982	0.144
**Overall survival in China**			
**ICERs, $**			
Per life-year	NA	NA	303,235
Per QALY	NA	NA	466,340
INHB, QALY, at threshold 38,258^a^	NA	NA	−1.532
INMB, $, at threshold 38,258^a^	NA	NA	−58,623
INHB, QALY, at threshold 84,866^a^	NA	NA	−0.616
INMB, $, at threshold 84,866^a^	NA	NA	−52,237
**Only progression-free survival in China**			
**ICERs, $**			
Per life-year	NA	NA	350,528
Per QALY	NA	NA	535,405
INHB, QALY, at threshold 38,258^a^	NA	NA	−1.550
INMB, $, at threshold 38,258^a^	NA	NA	−59,311
INHB, QALY, at threshold 84,866^a^	NA	NA	−0.634
INMB, $, at threshold 84,866^a^	NA	NA	−53,765
**Overall survival in US**			
**ICERs, $**			
Per life-year	NA	NA	669,489
Per QALY	NA	NA	976,925
INHB, QALY, at threshold 150,000^a^	NA	NA	−0.796
INMB, $, at threshold 150,000^a^	NA	NA	−119,400
**Only progression-free survival in US**			
**ICERs, $**			
Per life-year	NA	NA	773,903
Per QALY	NA	NA	1,118,607
INHB, QALY, at threshold 150,000^a^	NA	NA	−0.814
INMB, $, at threshold 150,000^a^	NA	NA	−122,100

Abbreviations: ICER, incremental cost-effectiveness ratio; INHB, incremental net health benefit; INMB, incremental net monetary benefit; NA, not applicable; QALYs, quality-adjusted life years.

^a^Compared with chemotherapy.

^b^Nondrug cost includes the costs of adverse event management, best supportive care per patient, routine follow-up, terminal care, and drug administration.

### Sensitivity analysis

The results of the DSA are showed in S3 Fig. Pembrolizumab cost and PFS utility influenced the base case analysis results for China and the US. In this study, it was found that the pembrolizumab plus chemotherapy strategy would become more advantageous as the price of pembrolizumab declined. According to the results, all parameters fluctuated within their upper and lower limits. This illustrated that the results of base-case analysis were relatively stable.

**Fig 1:**
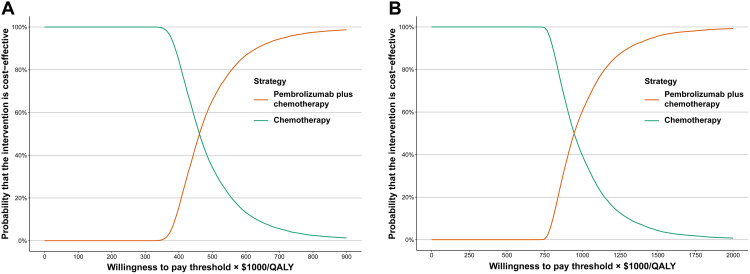
Probabilistic sensitivity analysis, cost-effectiveness acceptability curve. (A) cost-effectiveness acceptability curve from the perspective of the Chinese healthcare system; (B) cost-effectiveness acceptability curve from the perspective of the third-party payer in the United States.

Compared with the chemotherapy, the cost-effectiveness acceptability curves revealed that the pembrolizumab plus chemotherapy related to 0 probabilities of cost-effectiveness at the WTP threshold of $84,866, and $150,000 in the China and US, respectively (Fig 1).

Furthermore, the price of pembrolizumab was evaluated in relation to the ICER of pembrolizumab plus chemotherapy versus chemotherapy alone. When the WTP threshold was set at $38,258/QALY or $84,866/QALY, and the unit cost of pembrolizumab was below $2.44 or $5.08 per mg, respectively, the combination therapy could be considered cost-effective from the healthcare system perspective in China (S4 Fig). Moreover, when the WTP threshold was set at $150,000/QALY and the cost of pembrolizumab was below $6.72 per mg, the combination therapy could be considered cost-effective from the perspective of third-party payers in the US.

### Subgroup analysis

As part of our subgroup analysis, we varied the HR of OS between $38,258/QALY and $84,866/QALY in China, and $150,000/QALY in the US. Table 3 summarizes the results of the subgroup analysis. Pembrolizumab plus chemotherapy performed better when death risks were lower in most subgroups under all three price scenarios. However, there was no probability of cost-effectiveness in all the subgroups when the thresholds were equal to $38,258/QALY and $84,866/QALY in China, and $150,000 in the US.

**Table 3:** Summary of subgroup analyses obtained by varying the hazard ratios for overall survival.

Subgroup	Unstratified hazard ratio (95% CI)	China					USA			
		Change in cost, $^a^	Change in QALYs^a^	ICER, $/QALY	INHB		Change in cost, $^a^	Change in QALYs^a^	ICER, $/QALY	INHB, WTP of $150,000/QALY
					WTP of $38,258/QALY	WTP of $84,866/QALY				
**Age (years)**										
< 65	0.88 (0.73 to 1.05)	63,291	0.133	475,920	−1.521	−0.613	137,403	0.142	966,338	−0.774
≥ 65	0.79 (0.65 to 0.97)	64,632	0.224	289,018	−1.466	−0.538	150,405	0.239	630,256	−0.764
**Sex**										
Female	0.85 (0.70 to 1.03)	63,726	0.163	390,114	−1.502	−0.588	141,685	0.175	811,641	−0.770
Male	0.83 (0.69 to 1.00)	64,023	0.184	348,862	−1.490	−0.571	144,569	0.196	737,468	−0.768
**Geographical region**										
Asia	0.88 (0.72 to 1.08)	63,291	0.133	475,920	−1.521	−0.613	137,403	0.142	966,338	−0.774
Not Asia	0.80 (0.67 to 0.96)	64,478	0.214	301,819	−1.472	−0.546	148,937	0.228	653,137	−0.765
**ECOG performance status**										
0	0.87 (0.71 to 1.07)	63,434	0.143	443,219	−1.515	−0.604	138,825	0.153	907,330	−0.772
1	0.84 (0.70 to 1.00)	63,874	0.173	368,267	−1.496	−0.579	143,124	0.185	772,338	−0.769
**Smoking status**										
Current	0.90 (0.58 to 1.40)	63,006	0.113	559,182	−1.534	−0.630	134,577	0.121	1,116,802	−0.777
Former	0.87 (0.70 to 1.09)	63,434	0.143	443,219	−1.515	−0.604	138,825	0.153	907,330	−0.772
Never	0.82 (0.68 to 0.98)	64,173	0.194	331,514	−1.484	−0.563	146,019	0.207	706,333	−0.767
**Antibiotic use within 1 month of study start**										
No	0.86 (0.71 to 1.05)	63,580	0.153	414,890	−1.509	−0.596	140,252	0.164	856,259	−0.771
Yes	0.81 (0.68 to 0.98)	64,325	0.204	315,916	−1.478	−0.554	147,475	0.217	678,373	−0.766
**Site of origin**										
Extrahepatic	0.99 (0.73 to 1.35)	61,784	0.021	2,959,243	−1.594	−0.707	122,136	0.022	5,508,491	−0.792
Gallbladder	0.96 (0.73 to 1.26)	62,181	0.052	1,206,796	−1.574	−0.681	126,232	0.055	2,292,888	−0.786
Intrahepatic	0.76 (0.64 to 0.91)	65,103	0.253	256,844	−1.448	−0.514	154,845	0.270	572,920	−0.762
**Disease status**										
Locally advanced	0.69 (0.45 to 1.06)	66,253	0.322	205,647	−1.410	−0.459	165,416	0.343	482,541	−0.760
Metastatic	0.85 (0.74 to 0.98)	63,726	0.163	390,114	−1.502	−0.588	141,685	0.175	811,641	−0.770
**Biliary stent or drain**										
No	0.85 (0.74 to 0.98)	63,726	0.163	390,114	−1.502	−0.588	141,685	0.175	811,641	−0.770
Yes	0.72 (0.43 to 1.19)	65,751	0.293	224,480	−1.426	−0.482	160,849	0.312	515,618	−0.760
**Previous chemotherapy**										
No	0.86 (0.75 to 0.99)	63,580	0.153	414,890	−1.509	−0.596	140,252	0.164	856,259	−0.771
Yes	0.66 (0.41 to 1.08)	66,769	0.351	190,149	−1.394	−0.436	170,042	0.373	455,545	−0.760
**PD-L1 combined positive score**										
< 1	0.66 (0.41 to 1.08)	66,769	0.351	190,149	−1.394	−0.436	170,042	0.373	455,545	−0.760
≥ 1	0.85 (0.72 to 1.00)	63,726	0.163	390,114	−1.502	−0.588	141,685	0.175	811,641	−0.770
Unknown	0.77 (0.51 to 1.18)	64,944	0.244	266,660	−1.454	−0.522	153,359	0.260	590,381	−0.763

Abbreviations: ECOG, Eastern Cooperative Oncology Group; ICER, incremental cost-effectiveness ratio; INHB, incremental net health benefit; PD-L1, programmed death-ligand 1; QALY, quality-adjusted life-year; WTP, willingness to pay.

^a^Change in cost and change in QALYs represent the results of pembrolizumab plus chemotherapy minus chemotherapy.

## Discussion

KEYNOTE-966 shed some light on the potential impact of pembrolizumab plus chemotherapy on clinical efficiency in advanced BTC. In this study, it has been demonstrated that costs have substantial impact on advanced BTC treatment. Value-based oncology is warranted in light of escalating healthcare costs. The limited price information made it difficult for physicians and patients to determine whether pembrolizumab was cost-effective, which therefore, required a cost-effectiveness analysis. At the current price in China, at WTP thresholds of $38,258/QALY and $84,866/QALY, we concluded that pembrolizumab plus chemotherapy was not cost-effective at the current price, with increased costs of $63,864 for improved efficacy of 0.137 QALYs. Considering the WTP threshold of $150,000/QALY, pembrolizumab plus chemotherapy would not be preferred in the US at this price, as it increases the overall cost of $141,000 while improving effectiveness by 0.144 QALYs. According to DSA and PSA results, this finding was robust. As a result of the DSA analysis, the ICERs for China and the US were highly sensitive to the cost of pembrolizumab and the utility of the PFS. The fluctuation in subsequent treatment proportions using pembrolizumab was significant in the base-case analysis because the cost of pembrolizumab is high. The results of our price simulations were stable according to PSA. Results of the subgroup analysis indicated a general trend towards more cost-effectiveness for subgroups with better survival, despite pembrolizumab plus chemotherapy not being cost-effective in all subgroups.

We conducted a literature review of published economic studies of different types of chemotherapy used in treating BTC patients, and three studies were evaluated. A previously published study evaluated the cost-effectiveness of pembrolizumab plus chemotherapy compared with chemotherapy alone as first-line treatment for patients with BTC from the perspective of the Chinese healthcare system. Similar results revealed that pembrolizumab plus chemotherapy for BTC does not appear to be a cost-effective approach compared with chemotherapy as a standalone therapy. However, the detailed survival functions and subgroup analyses were not performed. Two previous studies have evaluated durvalumab plus chemotherapy as a first-line treatment for BTC. Based on the perspectives of the US and Chinese payers, one study compared durvalumab plus chemotherapy with chemotherapy, and the results indicated that the ICERs for the durvalumab plus chemotherapy group were $367,608.51/QALY and $381,864.39/QALY for Chinese and US payers, respectively [53]. On the other hand, another study found that durvalumab plus chemotherapy generated a cost-effectiveness ratio of $159,644.70/QALY as compared with chemotherapy, based on the perspective of the Chinese healthcare system [40]. A study in Japan compared gemcitabine alone with gemcitabine in combination treatment for advanced BTC, and the results revealed that the ICER of gemcitabine and cisplatin was approximately 14 million yen per QALY gained [59].

Our study differs from these studies in a number of ways. Firstly, some key model inputs were obtained from published studies, such as utilities, best supportive care costs, and terminal care costs. In addition, some studies did not evaluate the second-line treatment. Second, different models were employed in the published studies. The area under the survival curve was used to estimate health state occupancy in a partitioned survival model. There is a significant difference between partitioned survival models and state transition models (STM) since partitioned survival models do not include a structural link between intermediate clinical endpoints (e.g., progression of disease) and survival. It is possible to develop partitioned survival models without gaining access to IPD by considering clinical trial endpoints directly. The outcome of extrapolations from partitioned survival models and STMs, however, will differ significantly. Survival model specifications strongly influence extrapolations from STMs [60]. OS extrapolation in a partitioned survival model is influenced by only OS evidence, not PFS, whereas model structure and estimate of transition probability have a significant influence on OS extrapolation in STMs [60].

To our knowledge, this study is the first to evaluate the cost-effectiveness of pembrolizumab plus chemotherapy as a first-line therapy for BTC from the perspective of Chinese or US payers. This study has significant implications. It may not be appropriate to recommend a cost-effective treatment for the entire BTC patients. Moreover, dosing regimens should be tailored to each individual patient. Patients with BTC are likely to benefit from pembrolizumab in terms of survival. There is evidence in this study that chemotherapy is cost-effective, which may aid in accelerating the process of listing in health insurance and promoting gemcitabine plus cisplatin for the therapy of BTC. Secondly, our sensitivity analysis showed that the results of this study were robust. For fitting and extrapolating the survival data, we examined flexible parametric models that are more accurate than standard survival models. Physicians, patients, and policymakers may benefit from economic information regarding subgroups. Furthermore, this study provides actionable policy insights by quantifying the price points at which pembrolizumab plus chemotherapy attains cost-effectiveness under varying WTP thresholds. In China, reducing the unit price to $2.44 or $5.08 per mg, depending on the threshold applied, would align the regimen with accepted value-for-money criteria from the healthcare system perspective. In the US, a price below $6.72 per mg would be required to meet the $150,000/QALY benchmark from the perspective of third-party payers. These findings support value-based pricing and targeted reimbursement negotiations as strategies to enhance patient access while maintaining fiscal sustainability. Lastly, in our subgroup analysis, pembrolizumab plus chemotherapy generally demonstrated more favorable outcomes in patient groups with lower mortality risk, consistent with the expectation that improved survival translates into greater QALY gains. However, under current pricing and WTP thresholds, no subgroup achieved cost-effectiveness in either China or the US. This suggests that, while certain subgroups may be closer to the cost-effectiveness frontier, substantial price reductions or enhanced clinical benefit would be required to cross the threshold. From a policy perspective, these findings underscore the potential value of targeted reimbursement strategies or patient selection approaches that prioritize those most likely to derive substantial survival benefit. Such strategies could improve the economic value of pembrolizumab in BTC while optimizing resource allocation within constrained healthcare budgets.

There are some limitations in the study. First, since we did not have direct data from head-to-head comparisons, we did not include other ICIs, such as durvalumab, which have demonstrated clinical benefits in the past. Second, our study may be biased as a result of the limitations of the original clinical trial. It may not be entirely true that KEYNOTE-966 is effective in Chinese patients since more than half of the included population was not Asian. Owe to the lack of subgroup results of PFS data, we were unable to conduct a subgroup analysis, which reduced the robustness of our results. Accordingly, only one agent was reported as the proportion of subsequent treatments, which could have resulted in biases in the calculation of subsequent treatment costs. For this study to be improved, additional clinical data are required. Third, it remains to be seen whether pembrolizumab in combination with chemotherapy for BTC is effective on a long-term basis. This model estimated long-term efficacy, subsequent treatment, and long-term efficacy with much information still unknown in this 39-month follow-up. Even though the model and parameters have been validated, there may be some uncertainty. In particular, the partitioned survival model required extrapolation of OS and PFS beyond the trial follow-up using fitted parametric survival functions. Although multiple alternative distributions were tested in sensitivity analyses and results were generally robust, uncertainty inevitably remains regarding survival projections in the absence of long-term observational data. Forth, it is important to note that the utilities used in the model were not estimated using KEYNOTE-966, but rather from other health utility surveys in patients with BTC. The cost-effectiveness analysis is also influenced by the assumption that patients in both groups had the same utility, which may have led to some bias in the results. In addition, no suitable real‑world evidence dataset was available to validate the extrapolated survival tails. This limitation should be acknowledged, as external validation against real‑world evidence would provide additional support for the selected extrapolation functions and improve the robustness of long‑term projections. Fifth, the grades 1 and 2 AEs were not considered. The findings in the DSA suggest that these limitations may not have a significant impact, as the costs and disutilities associated with AEs were relatively minor. In general clinical practice, these AEs cannot be ignored. Last, this study was conducted from the health care system and third-party payer perspectives in China and the US, and thus only direct medical costs were included. Societal costs, such as productivity loss, transportation, and caregiver time, were not considered due to data unavailability, which may lead to underestimation of the total economic burden.

## Conclusion

Based on the results of this economic evaluation, pembrolizumab plus chemotherapy would not be cost-effective at current prices with WTP thresholds of $38,258/QALY and $84,866/QALY in China, or $150,000/QALY in the US. Decision-makers can use these results to make informed drug pricing decisions. Results from this study can assist clinicians in making optimal decisions about the treatment of BTC. Future studies can use the research framework developed for this study as a reference. A greater amount of evidence is also needed concerning the impact on budgets and the affordability of health care for patients.

## Supporting information

S1 Fig Model fitting analysis. (DOCX)

S2 Fig Validation of reconstructed individual patient data. (DOCX)

S3 Fig Tornado diagram of one-way sensitivity analyses of pembrolizumab plus chemotherapy versus chemotherapy. (DOCX)

S4 Fig Varying cost of pembrolizumab and ICERs in patients with advanced biliary tract cancer. Graphs represent the ICERs of pembrolizumab compared with chemotherapy.
(DOCX)

S1 Table Estimated parameters and AIC and BIC values from each survival model. (DOCX)

S2 Table Unit Costs, proportions, and total costs of second‑line treatment regimens in China and the US. (DOCX)

S3 Table Associated costs and disutility of grade ≥ 3 treatment-related adverse events. (DOCX)
