# Supplementary material for: A cost-effectiveness analysis comparing pembrolizumab combined with chemotherapy versus chemotherapy alone for advanced biliary tract cancer: US and China perspectives
Source: PLoS One. 2026 Jan 22;21(1):e0341154. doi: 10.1371/journal.pone.0341154 (PMC12826477; doi:10.1371/journal.pone.0341154)

**S1 Fig.** Model fitting analysis

To obtain the best model fit, the following investigations were carried out using pembrolizumab plus chemotherapy or chemotherapy as the model fit baseline, respectively. Based on AIC and BIC (S1 Table).

(A) Model-fitted versus original Kaplan-Meier curves for pembrolizumab plus chemotherapy, Log-logistic model was used to fit the OS and Lognormal model was used to fit the PFS Kaplan-Meier for pembrolizumab plus chemotherapy.


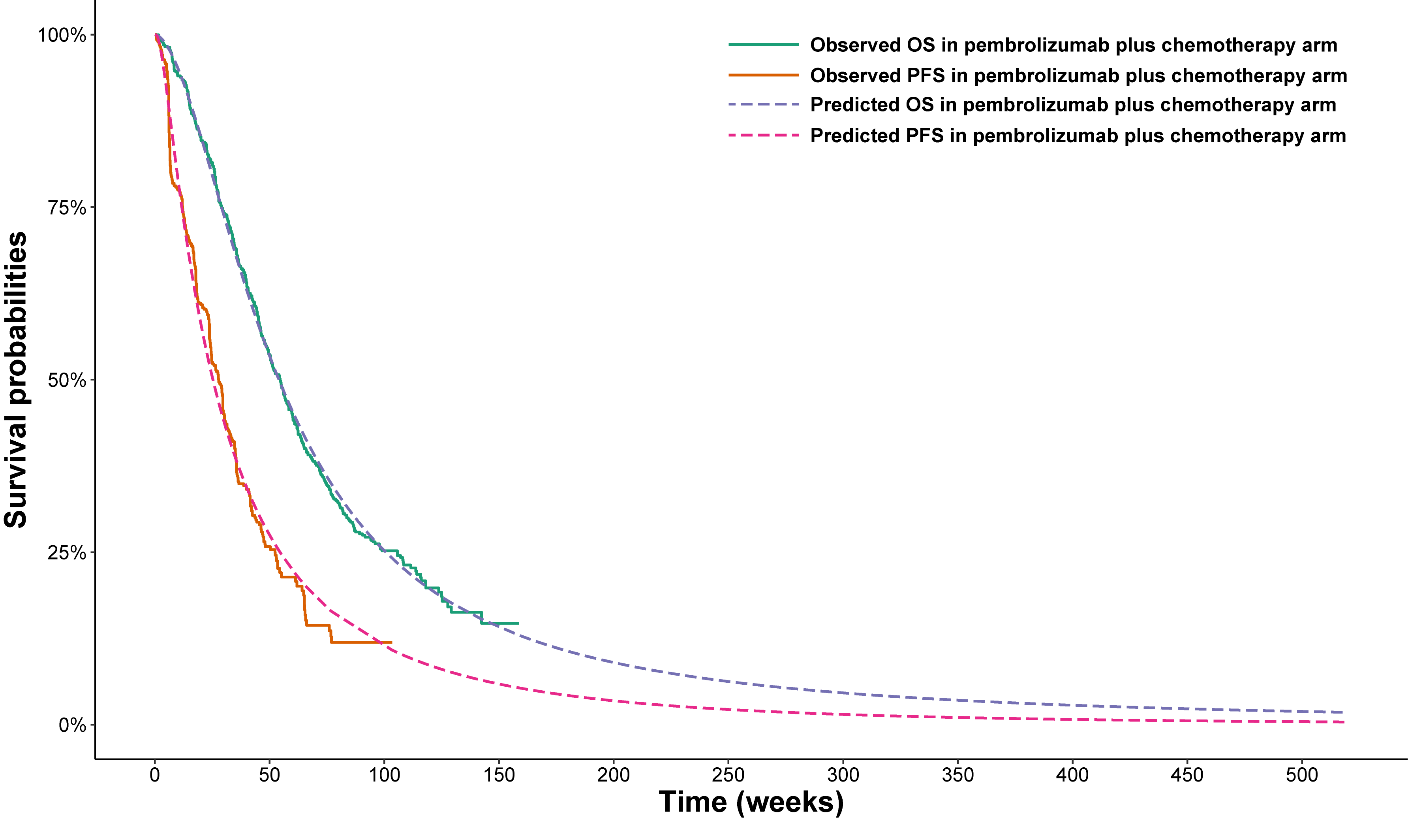


(B) Model-fitted versus original Kaplan-Meier curves for chemotherapy, Log-logistic model was used to fit the OS and Lognormal model was used to fit the PFS Kaplan-Meier for chemotherapy.


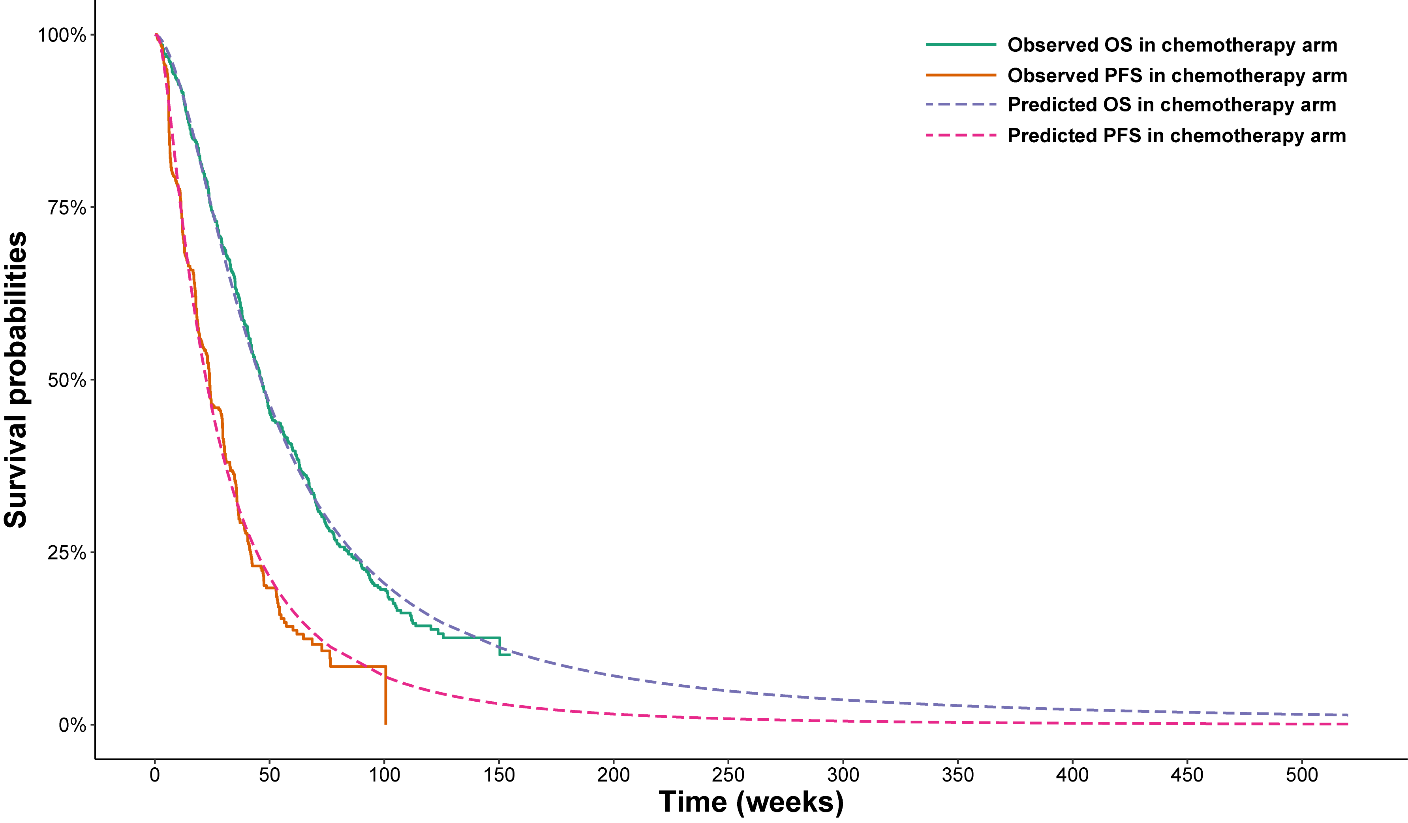

Supplement: S1 Fig — (DOCX) [file pone.0341154.s001.docx]
