# Supplementary material for: A cost-effectiveness analysis comparing pembrolizumab combined with chemotherapy versus chemotherapy alone for advanced biliary tract cancer: US and China perspectives
Source: PLoS One. 2026 Jan 22;21(1):e0341154. doi: 10.1371/journal.pone.0341154 (PMC12826477; doi:10.1371/journal.pone.0341154)

**S2 Fig.** Validation of reconstructed individual patient data. (A) Overall survival; (B) Progression-free survival


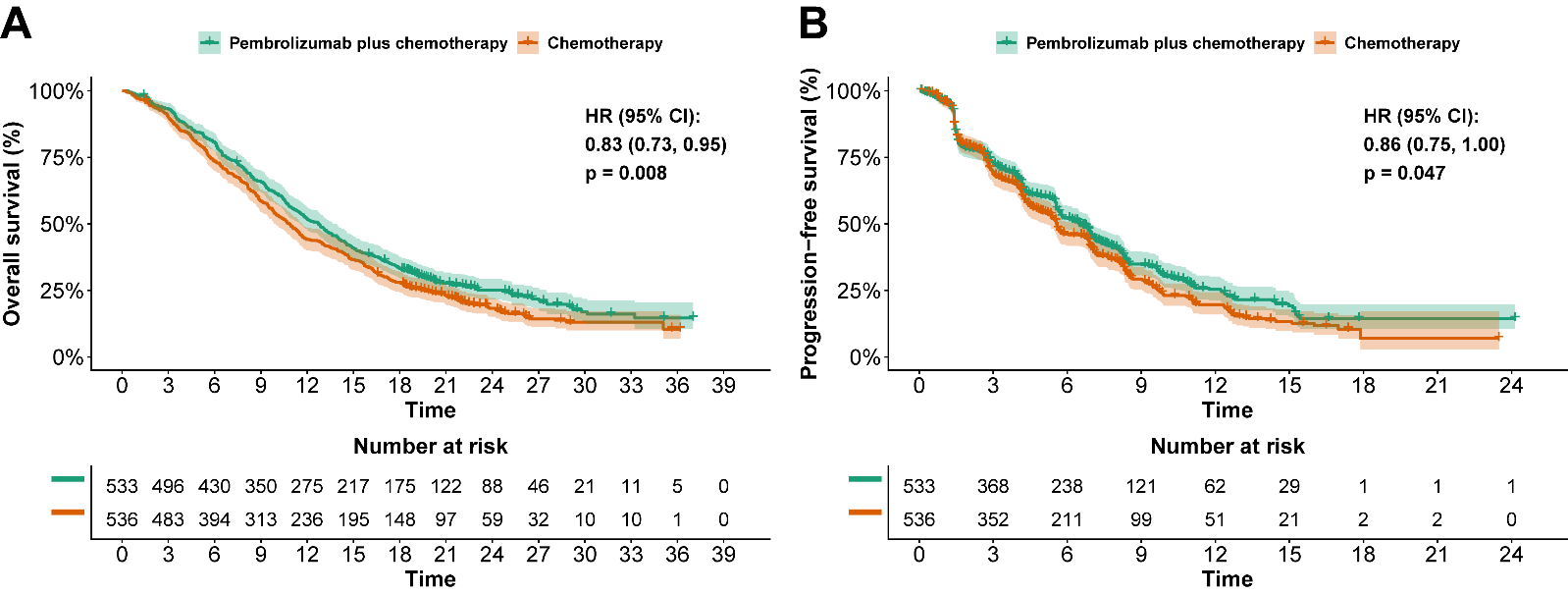

Supplement: S2 Fig — (DOCX) [file pone.0341154.s002.docx]
