# Supplementary material for: A cost-effectiveness analysis comparing pembrolizumab combined with chemotherapy versus chemotherapy alone for advanced biliary tract cancer: US and China perspectives
Source: PLoS One. 2026 Jan 22;21(1):e0341154. doi: 10.1371/journal.pone.0341154 (PMC12826477; doi:10.1371/journal.pone.0341154)

**S3 Fig.** Tornado diagram of one-way sensitivity analyses of pembrolizumab plus chemotherapy versus chemotherapy. (A) Tornado diagrams showing results of DSA (China); (B) Tornado diagrams showing results of DSA (United States).


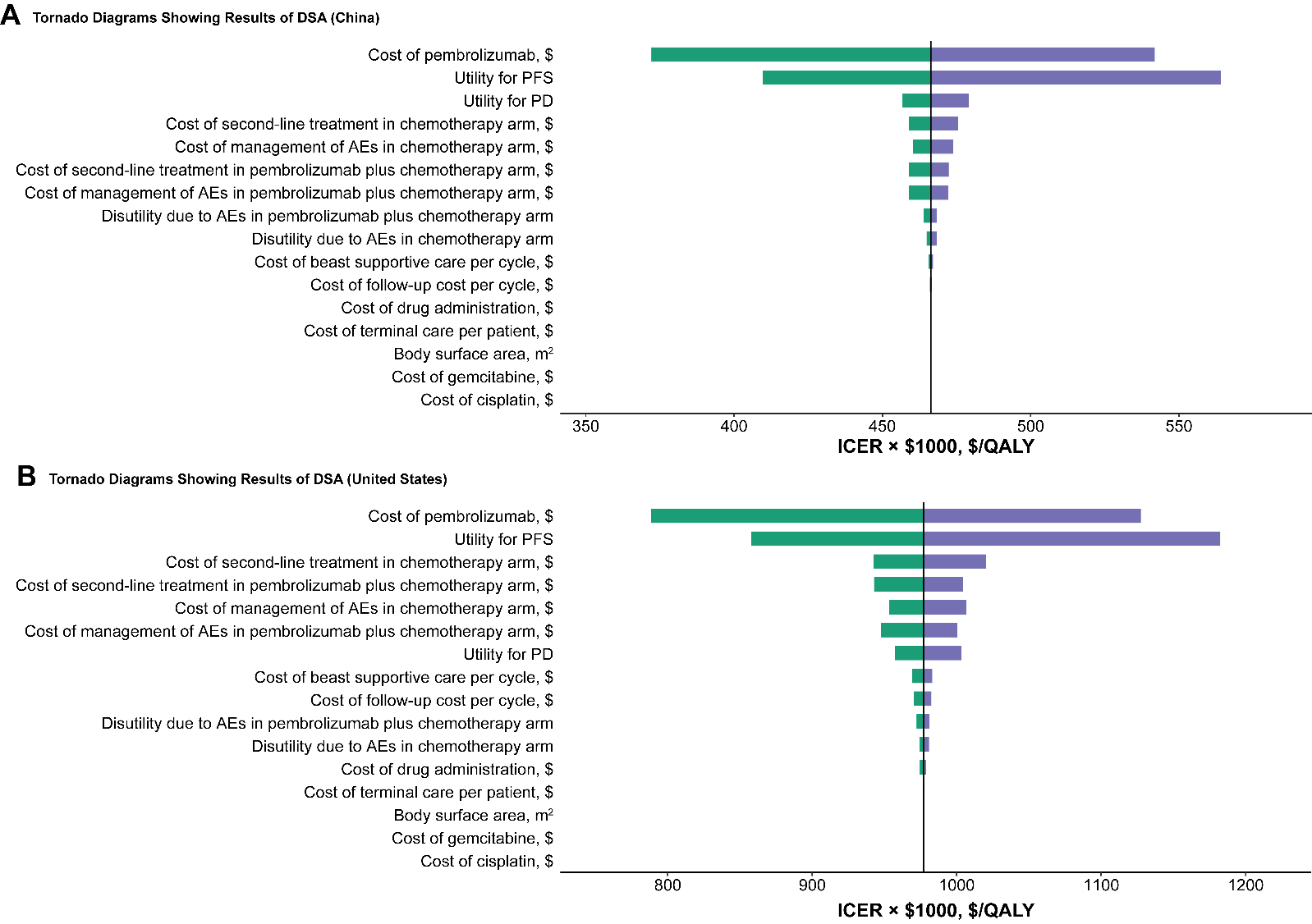

Supplement: S3 Fig — (DOCX) [file pone.0341154.s003.docx]
