# Supplementary material for: A cost-effectiveness analysis comparing pembrolizumab combined with chemotherapy versus chemotherapy alone for advanced biliary tract cancer: US and China perspectives
Source: PLoS One. 2026 Jan 22;21(1):e0341154. doi: 10.1371/journal.pone.0341154 (PMC12826477; doi:10.1371/journal.pone.0341154)

**S4 Fig.** Varying cost of pembrolizumab and ICERs in patients with advanced biliary tract cancer. Graphs represent the ICERs of pembrolizumab compared with chemotherapy. (A) from the perspective of the Chinese healthcare system; (B) from the perspective of the third-party payer in the United States.


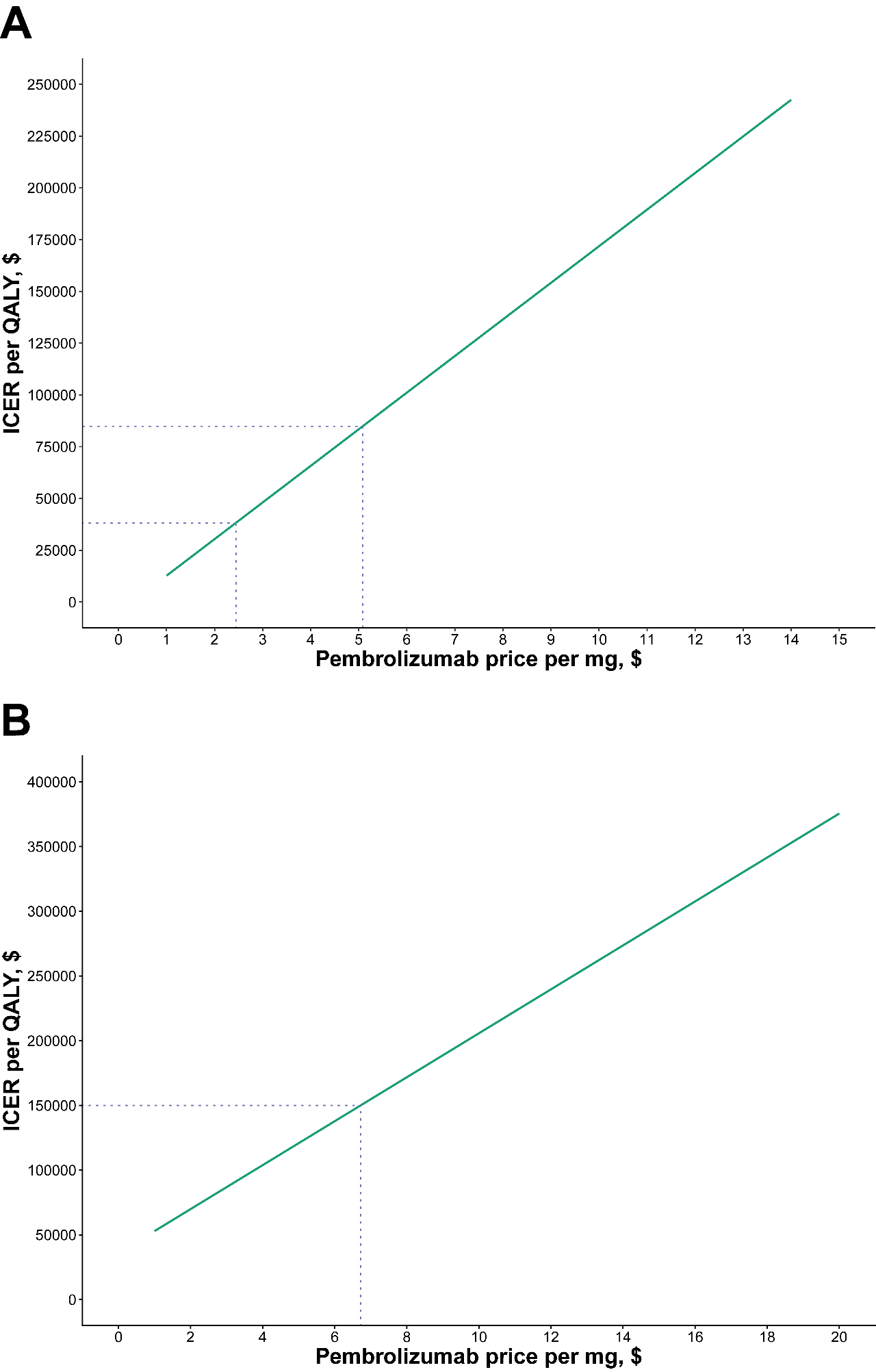

Supplement: S4 Fig — Graphs represent the ICERs of pembrolizumab compared with chemotherapy. (DOCX) [file pone.0341154.s004.docx]
