# Supplementary material for: A cost-effectiveness analysis comparing pembrolizumab combined with chemotherapy versus chemotherapy alone for advanced biliary tract cancer: US and China perspectives
Source: PLoS One. 2026 Jan 22;21(1):e0341154. doi: 10.1371/journal.pone.0341154 (PMC12826477; doi:10.1371/journal.pone.0341154)
Supplement: S1 Table — (DOCX) [file pone.0341154.s005.docx]

**S1 Table.** Estimated parameters and AIC and BIC values from each survival model.

| **Strategies** | **Distributions** | **Parameters** | **est** | **se** | **L95%** | **U95%** | **AIC** | **BIC** |
| --- | --- | --- | --- | --- | --- | --- | --- | --- |
| **Results of OS** | | | | | | | | |
| **Pembrolizumab plus chemotherapy** | Exponential | rate | 0.0128 | 0.0006 | 0.0117 | 0.0141 | 4404.395 | 4408.673 |
|  | Weibull | shape | 1.2908 | 0.0535 | 1.1902 | 1.4000 | 4372.45 | 4381.007 |
|  |  | scale | 0.0036 | 0.0009 | 0.0023 | 0.0058 |  |  |
|  | Gamma | shape | 1.5401 | 0.0978 | 1.3599 | 1.7442 | 4364.65 | 4373.207 |
|  |  | rate | 0.0212 | 0.0017 | 0.0181 | 0.0249 |  |  |
|  | Lognormal | meanlog | 3.9771 | 0.0453 | 3.8882 | 4.0659 | 4365.019 | 4373.576 |
|  |  | sdlog | 1.0044 | 0.0368 | 0.9348 | 1.0792 |  |  |
|  | Gompertz | shape | 0.0046 | 0.0015 | 0.0017 | 0.0074 | 4397.15 | 4405.707 |
|  |  | rate | 0.0105 | 0.0009 | 0.0089 | 0.0124 |  |  |
|  | Log-logistic | shape | 1.7679 | 0.0736 | 1.6294 | 1.9183 | **4352.533** | **4361.09** |
|  |  | scale | 54.0860 | 2.3108 | 49.7414 | 58.8102 |  |  |
|  | Generalized gamma | mu | 4.1378 | 0.0693 | 4.0019 | 4.2736 | 4359.11 | 4371.946 |
|  |  | sigma | 0.9144 | 0.0483 | 0.8245 | 1.0142 |  |  |
|  |  | Q | 0.4051 | 0.1443 | 0.1223 | 0.6879 |  |  |
| **Chemotherapy** | Exponential | rate | 0.0152 | 0.0007 | 0.0139 | 0.0167 | 4585.614 | 4589.898 |
|  | Weibull | shape | 1.2679 | 0.0501 | 1.1735 | 1.3700 | 4555.127 | 4563.695 |
|  |  | scale | 0.0049 | 0.0011 | 0.0032 | 0.0075 |  |  |
|  | Gamma | shape | 1.4867 | 0.0909 | 1.3188 | 1.6759 | 4549.44 | 4558.008 |
|  |  | rate | 0.0238 | 0.0018 | 0.0204 | 0.0277 |  |  |
|  | Lognormal | meanlog | 3.8039 | 0.0453 | 3.7150 | 3.8928 | 4565.916 | 4574.484 |
|  |  | sdlog | 1.0224 | 0.0357 | 0.9547 | 1.0948 |  |  |
|  | Gompertz | shape | 0.0050 | 0.0015 | 0.0021 | 0.0079 | 4576.614 | 4585.182 |
|  |  | rate | 0.0124 | 0.0010 | 0.0106 | 0.0145 |  |  |
|  | Log-logistic | shape | 1.7565 | 0.0703 | 1.6240 | 1.8998 | **4546.565** | **4555.133** |
|  |  | scale | 46.2016 | 1.9683 | 42.5004 | 50.2250 |  |  |
|  | Generalized gamma | mu | 4.0436 | 0.0660 | 3.9144 | 4.1729 | 4548.279 | 4561.131 |
|  |  | sigma | 0.8861 | 0.0457 | 0.8009 | 0.9804 |  |  |
|  |  | Q | 0.5800 | 0.1336 | 0.3181 | 0.8419 |  |  |
| **Results of PFS** | | | | | | | | |
| **Pembrolizumab plus chemotherapy** | Exponential | rate | 0.0258 | 0.0014 | 0.0233 | 0.0286 | 3327.012 | 3331.29 |
|  | Weibull | shape | 1.1623 | 0.0506 | 1.0672 | 1.2658 | 3317.866 | 3326.423 |
|  |  | scale | 0.0145 | 0.0027 | 0.0100 | 0.0210 |  |  |
|  | Gamma | shape | 1.2908 | 0.0832 | 1.1376 | 1.4647 | 3314.253 | 3322.81 |
|  |  | rate | 0.0354 | 0.0032 | 0.0296 | 0.0423 |  |  |
|  | Lognormal | meanlog | 3.2319 | 0.0540 | 3.1260 | 3.3377 | **3307.575** | **3320.411** |
|  |  | sdlog | 1.1380 | 0.0444 | 1.0542 | 1.2286 |  |  |
|  | Gompertz | shape | 0.0047 | 0.0032 | -0.0016 | 0.0109 | 3326.908 | 3335.465 |
|  |  | rate | 0.0235 | 0.0020 | 0.0199 | 0.0277 |  |  |
|  | Log-logistic | shape | 1.5220 | 0.0667 | 1.3968 | 1.6584 | 3311.497 | 3320.054 |
|  |  | scale | 25.7441 | 1.3431 | 23.2417 | 28.5159 |  |  |
|  | Generalized gamma | mu | 3.3852 | 0.0893 | 3.2101 | 3.5603 | 3309.729 | 3318.286 |
|  |  | sigma | 1.0490 | 0.0632 | 0.9321 | 1.1804 |  |  |
|  |  | Q | 0.3515 | 0.1735 | 0.0114 | 0.6916 |  |  |
| **Chemotherapy** | Exponential | rate | 0.0297 | 0.0015 | 0.0269 | 0.0328 | 3479.313 | 3483.597 |
|  | Weibull | shape | 1.2319 | 0.0504 | 1.1369 | 1.3348 | 3457.827 | 3466.395 |
|  |  | scale | 0.0132 | 0.0025 | 0.0092 | 0.0190 |  |  |
|  | Gamma | shape | 1.4475 | 0.0916 | 1.2787 | 1.6386 | 3450.046 | 3458.614 |
|  |  | rate | 0.0461 | 0.0039 | 0.0391 | 0.0544 |  |  |
|  | Lognormal | meanlog | 3.1082 | 0.0470 | 3.0160 | 3.2004 | **3433.896** | **3442.464** |
|  |  | sdlog | 1.0149 | 0.0379 | 0.9433 | 1.0919 |  |  |
|  | Gompertz | shape | 0.0067 | 0.0030 | 0.0008 | 0.0126 | 3476.636 | 3485.204 |
|  |  | rate | 0.0261 | 0.0021 | 0.0223 | 0.0305 |  |  |
|  | Log-logistic | shape | 1.6814 | 0.0704 | 1.5488 | 1.8253 | 3442.654 | 3451.222 |
|  |  | scale | 22.6739 | 1.0594 | 20.6896 | 24.8484 |  |  |
|  | Generalized gamma | mu | 3.1643 | 0.0859 | 2.9960 | 3.3325 | 3435.305 | 3448.157 |
|  |  | sigma | 0.9939 | 0.0471 | 0.9057 | 1.0907 |  |  |
|  |  | Q | 0.1316 | 0.1710 | -0.2036 | 0.4667 |  |  |
