# Supplementary material for: A cost-effectiveness analysis comparing pembrolizumab combined with chemotherapy versus chemotherapy alone for advanced biliary tract cancer: US and China perspectives
Source: PLoS One. 2026 Jan 22;21(1):e0341154. doi: 10.1371/journal.pone.0341154 (PMC12826477; doi:10.1371/journal.pone.0341154)
Supplement: S2 Table — (DOCX) [file pone.0341154.s006.docx]

**S2 Table.** Unit Costs, proportions, and total costs of second‑line treatment regimens in China and the US.

| Second-line treatment plan | China | | | US | | |
| --- | --- | --- | --- | --- | --- | --- |
|  | Cost | Proportion and total cost | | Cost | Proportion and total cost | |
|  |  | Pembrolizumab plus chemotherapy | Chemotherapy | | Pembrolizumab plus chemotherapy | Chemotherapy |
| FOLFOX6 |  |  |  |  |  |  |
| Oxaliplatin, $/1 mg | 0.48 |  |  | 0.15 |  |  |
| Folinic acid, $/1 mg | 1.01 |  |  | 0.24 |  |  |
| Fluorouracil, $/1 mg | 0.07 |  |  | 0.0047 |  |  |
| FOLFOX6 plan Total, $ | 104.12 | 0.43 | 0.43 | 55.46 | 0.43 | 0.43 |
| Immune checkpoint inhibitor |  |  |  |  |  |  |
| Nivolumab, $/1 mg | 13.76 |  |  | 29.91 |  |  |
| Pembrolizumab, $/1 mg | 26.66 |  |  | 55.42 |  |  |
| Immune checkpoint inhibitor plan total, $ | 1714.68 | 0.05 | 0.07 | 3641.89 | 0.05 | 0.07 |
| Targeted therapy |  |  |  |  |  |  |
| Entrectinib, $/1 mg | 0.31 |  |  | 2.55 |  |  |
| Targeted therapy plan total, $ | 1284.72 | 0.09 | 0.12 | 10689.42 | 0.09 | 0.12 |
| Second-line treatment plan total |  | 246.13 | 318.97 |  | 1167.99 | 1561.51 |
