# Supplementary material for: A cost-effectiveness analysis comparing pembrolizumab combined with chemotherapy versus chemotherapy alone for advanced biliary tract cancer: US and China perspectives
Source: PLoS One. 2026 Jan 22;21(1):e0341154. doi: 10.1371/journal.pone.0341154 (PMC12826477; doi:10.1371/journal.pone.0341154)
Supplement: S3 Table — (DOCX) [file pone.0341154.s007.docx]

**S3 Table.** Associated costs and disutility of grade ≥ 3 treatment-related adverse events.

| **Adverse Event^a^** | **No. of patients (%)^b^** | **Costs in United States** | | **Costs in China** | | **Disutility** | **Reference** |
| --- | --- | --- | --- | --- | --- | --- | --- |
|  |  | **Costs in 2023 USD** | **Reference** | **Costs in 2023 USD** | **Reference** |  |  |
| **Pembrolizumab plus chemotherapy** | |  |  |  |  |  |  |
| Neutropenia | 451 (85%) | 20,276 | Wong et al., 2018 | 3,580 | Wu et al., 2012 | 0.348 | Nafees et al., 2017 |
| Anaemia | 152 (29%) | 8,802 | Konidaris et al., 2021 | 4,124 | Wu et al., 2012 | 0.072 | Freeman et al., 2015 |
| Thrombocytopenia | 94 (18%) | 4,937 | Liou et al., 2007 | 4,175 | Wu et al., 2012 | 0.108 | Konidaris et al., 2021 |
| Fatigue, asthenia | 37 (7%) | 171 | Liou et al., 2007 | 125 | Hou et al., 2020 | 0.288 | Nafees et al., 2017 |
| Decreased white blood cell count | 61 (12%) | 5,300 | Wong et al., 2018 | 116 | Shao et al., 2022 | 0.200 | Nafees et al., 2017 |
| Weighted average^c^ |  | 21,316 |  | 5,001 |  | 0.043 |  |
| **Chemotherapy** |  |  |  |  |  |  |  |
| Neutropenia | 451 (85%) | 20,276 | Wong et al., 2018 | 3,580 | Wu et al., 2012 | 0.348 | Nafees et al., 2017 |
| Anaemia | 154 (29%) | 8,802 | Konidaris et al., 2021 | 4,124 | Wu et al., 2012 | 0.072 | Freeman et al., 2015 |
| Thrombocytopenia | 107 (20%) | 4,937 | Liou et al., 2007 | 4,175 | Wu et al., 2012 | 0.108 | Konidaris et al., 2021 |
| Fatigue, asthenia | 39 (7%) | 171 | Liou et al., 2007 | 125 | Hou et al., 2020 | 0.288 | Nafees et al., 2017 |
| Decreased white blood cell count | 47 (9%) | 5,300 | Wong et al., 2018 | 116 | Shao et al., 2022 | 0.200 | Nafees et al., 2017 |
| Weighted average^c^ |  | 21,331 |  | 5,116 |  | 0.039 |  |

^a^Our analysis only included and evaluated grade ≥ 3 treatment-related adverse events.

^b^Number within treatment arm: pembrolizumab plus chemotherapy (N = 533), chemotherapy (N = 536).

^c^Calculated as an average cost of toxicity using the weighted frequency of occurrence. This value was used in the base-case model.

**References**

Freeman K, Connock M, Cummins E, Gurung T, Taylor-Phillips S, Court R, et al. Fluorouracil Plasma Monitoring: Systematic Review and Economic Evaluation of the My5-Fu Assay for Guiding Dose Adjustment in Patients Receiving Fluorouracil Chemotherapy by Continuous Infusion. *Health Technol Assess* (2015) 19(91):1-321, v-vi. doi: 10.3310/hta19910

Hou Y, Wu B. Atezolizumab plus bevacizumab versus sorafenib as first-line treatment for unresectable hepatocellular carcinoma: a cost-effectiveness analysis. *Cancer Commun* (2020) 40(12):743–5. doi: 10.1002/cac2.12110

Konidaris G, Paul E, Kuznik A, Keeping S, Chen CI, Sasane M, et al. Assessing the Value of Cemiplimab for Adults with Advanced Cutaneous Squamous Cell Carcinoma: A Cost-Effectiveness Analysis. *Value Health* (2021) 24(3):377-87. doi: 10.1016/j.jval.2020.09.014

Liou SY, Stephens JM, Carpiuc KT, Feng W, Botteman MF, Hay JW. Economic burden of haematological adverse effects in cancer patients: a systematic review. *Clin Drug Investig* (2007) 27(6):381–96. doi: 10.2165/00044011-200727060-00002

Nafees B, Lloyd AJ, Dewilde S, Rajan N, Lorenzo M. Health State Utilities in Non-Small Cell Lung Cancer: An International Study. *Asia Pac J Clin Oncol* (2017) 13(5):e195-e203. doi: 10.1111/ajco.12477

Shao T, Zhao M, Tang, W. Cost-effectiveness analysis of sintilimab vs. placebo in combination with chemotherapy as first-line therapy for local advanced or metastatic oesophageal squamous cell carcinoma. *Front Oncol* (2022) 12:953671. doi: 10.3389/fonc.2022.953671

Wong W, Yim YM, Kim A, Cloutier M, Gauthier-Loiselle M, Gagnon-Sanschagrin P, et al. Assessment of Costs Associated with Adverse Events in Patients with Cancer. *PLoS One* (2018) 13(4):e0196007. doi: 10.1371/journal.pone.0196007

Wu B, Dong B, Xu Y, Zhang Q, Shen J, Chen H, et al. Economic Evaluation of First-Line Treatments for Metastatic Renal Cell Carcinoma: A Cost-Effectiveness Analysis in a Health Resource-Limited Setting. *PLoS One* (2012) 7(3):e32530. doi: 10.1371/journal.pone.0032530
